# Accurate Chemogenetics Determines Electroacupuncture Analgesia Through Increased CB1 to Suppress the TRPV1 Pathway in a Mouse Model of Fibromyalgia

**DOI:** 10.3390/life15050819

**Published:** 2025-05-20

**Authors:** Huan-Chin Lin, Hi-Joon Park, Hsien-Yin Liao, Kai-Ting Chuang, Yi-Wen Lin

**Affiliations:** 1 College of Chinese Medicine, Graduate Institute of Acupuncture Science, China Medical University, Taichung 404328, Taiwan; peishiuan1010@gmail.com; 2 Department of Traditional Chinese Medicine, Feng Yuan Hospital, Ministry of Health and Welfare, Taichung 420255, Taiwan; 3 Acupuncture and Meridian Science Research Center, Kyung Hee University, Seoul 02447, Republic of Korea; acufind@khu.ac; 4 College of Chinese Medicine, School of Post-Baccalaureate Chinese Medicine, China Medical University, Taichung 404328, Taiwan; 017215@tool.caaumed.org.tw; 5 Department of Family Medicine, China Medical University Hsinchu Hospital, China Medical University, Hsinchu 302056, Taiwan; 6 Chinese Medicine Research Center, China Medical University, Taichung 404328, Taiwan

**Keywords:** electroacupuncture, fibromyalgia, CB1, TRPV1, chemogenetics, PAG

## Abstract

Fibromyalgia, one of the most challenging pains to treat, lacks impartial considerations for diagnosis and useful assessment. The core symptoms are persistent extensive pain accompanied by fatigue, psychological disorders, sleep disturbance, and obesity. This study aims to explore the role of cannabinoid receptor 1 (CB1) on transient receptor potential V1 (TRPV1) signaling pathways in a mouse model of fibromyalgia. This model was subjected to intermittent cold stress (ICS) to induce fibromyalgia, as measured by the nociceptive behavior determined by von Frey and Hargreaves’ tests. Our results showed a lower mechanical threshold (2.32 ± 0.12 g) and thermal latency (4.14 ± 0.26 s) in ICS-induced fibromyalgia mice. The hyperalgesia could be alleviated by 2 Hz electroacupuncture (EA) or by TRPV1 knockout. We found decreased CB1 receptors, upregulated TRPV1, and related kinases in the dorsal root ganglion, spinal cord, hypothalamus, and periaqueductal gray in fibromyalgia mice. EA reversed these effects associated with fibromyalgia, aligning with observations in *Trpv1*^−/−^ mice. Peripheral acupoint or the intracerebral ventricle injection of a CB1 agonist significantly attenuated mechanical and thermal hyperalgesia. The EA analgesic effect was reversed by a CB1 antagonist injection, suggesting the involvement of the CB1 signaling pathway. The accurate chemogenetic activation of paraventricular nucleus (PVN), which is a structure of the hypothalamus, initiated fibromyalgia pain. The chemogenetic inhibition of PVN attenuated fibromyalgia pain via the downregulation of TRPV1 pathway. Our discoveries shed light on the involvement of CB1 in the TRPV1 signaling pathway in the effects of EA in fibromyalgia, suggesting its potential as a treatment target.

## 1. Introduction

Fibromyalgia is a chronic condition characterized by widespread pain affecting multiple body sites, including arms, legs, head, chest, abdomen, and back. Patients often describe the pain as aching, burning, or sore. It frequently coexists with tension headaches, irritable bowel syndrome, anxiety, and depression, particularly in women [1]. Because there is no cure for fibromyalgia pain so far, current medications can only manage the symptoms. Nonpharmacological approaches such as nutrients, exercise, meditation, sleep therapy, yoga, and relaxation may provide additional benefits. The underlying mechanisms of fibromyalgia are thought to involve central sensitization due to repeated nerve stimulation, the overexpression of neurotransmitters, or neuromodulators in the brain [2]. These phenomena may result from genetic mutations, infections, or psychological stress. Fibromyalgia affects about 2–8% of the population [1,3]. The U.S. Food and Drug Administration (FDA)-approved medications for fibromyalgia include duloxetine (Cymbalta), milnacipran (Savella), and pregabalin (Lyrica). Additionally, gabapentinoids, sedatives, selective serotonin reuptake inhibitors, serotonin norepinephrine reuptake inhibitors, and tricyclic compounds are used, though they are associated with several side effects. Given its chronic nature and treatment challenges, fibromyalgia imposes a high economic burden [4,5,6].

Acupuncture, a main component of traditional Chinese medicine, involves inserting steel needles into specific acupoints on the body and widely reported to relieve pain. According to Chinese medicine theory, acupuncture regulates the meridians to control qi and balance the energy flow within the body. Advances in neuroanatomy have demonstrated that acupoints are specific regions which can easily stimulate connective tissue, muscle, and peripheral nerves for therapeutic purposes. Recent studies have shown that electroacupuncture (EA) or the optogenetic stimulation of nerve terminals at the ST36 acupoint, can activate the vagal–adrenal axis to produce anti-inflammatory effects in mouse models of sepsis [7,8]. Additionally, we previously demonstrated that EA triggers the local release of adenosine triphosphate, interleukin-1β, interleukin-6, glutamate, substance P, and histamine [9]. Further, EA has been reported to reduce inflammatory, neuropathic, and fibromyalgia pain in various mouse models [10,11,12,13,14,15,16]. Specifically, EA decreased fibromyalgia pain by reducing the levels of circulating inflammatory cytokines such as interleukins, TNF-α, and IFN-γ in mice [17]. Despite the promising findings, the precise mechanisms by which EA exerts its effects remain incompletely understood.

Cannabinoid (CB) receptors are 7-transmembrane domain receptors that can be activated at both peripheral or central sites with the cannabis plant. CB receptors are considered G-protein-coupled receptors with two distinguished subtypes: CB1 and CB2 receptors, encoded by *CNR1* and *CNR2*, respectively. Cannabinoids can bind at different receptors such as CB1 in neurons and CB2 in immune cells. CB1 is mostly found in the brain, especially in the hippocampus, cerebellum, hypothalamus (Hypo), amygdala, periaqueductal gray (PAG), and cerebral cortex [18,19]. Endocannabinod ligands such as 2-arachidonoylglycerol (2-AG) and arachidonylethanolamide (AEA) can bind at presynaptic CB1 receptors to inhibit adenylate cyclase, cAMP, A-type K+ channels, and voltage-gated Ca^2+^ channels to reduce synaptic transmission. CB receptors have been implicated in physiological and pathophysiological conditions involved in the regulation of mood, appetite, pain sensation, and immune response [20,21]. The sequential signaling possessions of CB1 were next modified by Src homology 3-domain growth factor receptor-bound 2-like endophilin interacting protein 1 (SGIP1), which can decrease ERK1/2 signaling. In addition, CB1-selective inhibitors were used for weight reduction and smoking cessation [22].

On the other hand, transient receptor potential V1 (TRPV1) has been implicated in inflammation, cancer, and immunity progress. TRPV1 has been used as a target of drugs for human pain conditions. However, the identification of new targets for TRPV1 structure, agonists, and mechanisms is urgently required [23,24]. TRPV1 activation cooperates with protein kinase A (PKA), phosphoinositide 3-kinase (PI3K), and PKC in the modulation of pain, indicating its crucial role in central sensitization linked with fibromyalgia pain at both peripheral and central nervous systems [25,26]. The epsilon isoform of protein kinase C (PKCɛ) is used as a transient insult to deliver hyperalgesia in nociceptors to form long-lasting central sensitization changes, a well-known factor involved in fibromyalgia pain. MAPK is considered to be involved in the inflammation and pain signaling pathway including extracellular signal-regulated protein kinase (ERK), p38, and c-Jun N-terminal kinase/stress-activated protein kinase (JNK) [27,28]. These components have been implicated in nociceptive processes associated with painful sensation, neuronal plasticity, central sensitization and certain cognitive function [28,29]. Further, the PI3K-Akt-mTOR signaling pathway is involved in changes in both peripheral and central nociceptive response for central sensitization. Moreover, nuclear factor kappa-light-chain-enhancer of activated B cells (NF-kB) seems to be involved in fibromyalgia pain development, while its suppression appears to have therapeutic benefits for pain [30,31].

In the current research, our hypothesis was that EA could diminish fibromyalgia symptoms via CB1 signaling. In addition, novel and detailed cellular mechanisms behind the pain-relieving properties of EA to treat fibromyalgia pain would be obtained. We found that hyperalgesia increased the expression of TRPV1 and related kinases in a mouse model of fibromyalgia pain. Such effects were observed in the mouse dorsal root ganglion (DRG), spinal cord dorsal horn (SCDH), hypothalamus (Hypo), and periaqueductal gray (PAG). These effects were reversed by EA treatment, even in *Trpv1*^−/−^ mice. Further, peripheral acupoint or intracerebral ventricle injection of a CB1 agonist significantly attenuated mechanical and thermal hyperalgesia. The EA analgesic effect was reversed by a CB1 antagonist injection suggesting the involvement of CB1 signaling pathway. In addition, intracerebral ventricle injection of a CB1 agonist or antagonist significantly attenuated hyperalgesia through central, but not peripheral CB1 signaling. The chemogenetic activation of paraventricular nucleus (PVN) initiated fibromyalgia pain. The chemogenetic inhibition of PVN attenuated fibromyalgia pain via the downregulation of TRPV1 pathway. Based on the above, these data provide a novel indication for EA in mice fibromyalgia pain.

## 2. Materials and Methods

### 2.1. Animals

The experimental subjects were 8–12-week-old female C57B/L6 wild-type mice (18–20 g)—we used female mice to reflect the higher prevalence of fibromyalgia pain in human females than males—bought from BioLasc Taiwan Ltd. (Yilan, Taiwan). After their arrival, mice were placed in a home cage under a 12 h light and dark cycle, with the light conditions being from 6 a.m. to 6 p.m., at 25 °C and 60% humidity. We used the G*Power 3.1.9.7 statistic software program to determine the sample size. Nine mice per group was considered to be the minimum number required for a significance alpha level of 0.05 and a power of 80%. The mouse experiments were approved by the Institute of Animal Care and Use Committee of China Medical University (permit no. CMUIACUC-2023-071), Taiwan, following the Guide for the use of Laboratory Animals (National Academy Press, Taipei, Taiwan).

### 2.2. Mouse Fibromyalgia Pain Induction

Mice were assigned at random to one of four groups, including normal (Normal), ICS-induced fibromyalgia pain (FM), ICS-induced fibromyalgia pain with EA (FM + EA), and ICS-induced fibromyalgia pain in *Trpv1^−/−^* mice (FM + *Trpv1^−/−^*). To induce mice fibromyalgia pain, mice in the fibromyalgia groups were placed into a 4 °C environment, while the normal group experienced a maintained normal temperature. At 10 a.m. the following day, the fibromyalgia groups were relocated to the normal environment at 25 °C for 30 min, then back to 4 °C for 30 min. This process was carried out until 4 p.m. to a total of 6 h, before they were repositioned again overnight from 4 p.m., and repeated for the first 3 days.

### 2.3. Electroacupuncture

After anesthesia with 5% isoflurane for induction, the mice were placed in 1% gas for maintenance via a head-stuffed tube for inhalation. A pair of 1-inch-long acupuncture needles (32G, Yu Kuang Chem. Ind. Corp., Tainan City, Taiwan) were inserted bilaterally into the mouse ST36 acupoint. The murine ST36 is found 3–4 mm below the patella, between the fibula and tibia, and on the anterior side of the anterior tibial muscle. In addition, electrical stimulation was delivered through a constant square pulse at a depth of 3–4 mm with 1 mA intensity, 2 Hz frequency, and 150 μs width for 20 min, utilizing an electronic source from a Trio 300 stimulator (Ito, Tokyo, Japan). The EA treatment sessions were conducted on day 3 and day 4.

### 2.4. Pain Behavior Test

Mechanical and thermal nociceptive behaviors were evaluated 3 times, on days 0, 3, and 4, before and after fibromyalgia pain induction. Mice were enclosed in Plexiglas boxes (9.5 × 11 × 6.5 cm^3^) above a steel mesh in a dark, noiseless, room maintained at room temperature to allow them to adapt to their new surroundings. When mice were not sleeping, scratching, or grooming, the von Frey test was performed three times per section, separated by 10 min (IITC Life Science Inc., Woodland Hills, CA, USA). Then, the Hargreaves test was applied to measure mouse thermal responses. Mice were separated into various Plexiglas enclosures to restrict interaction. After 30 min of environmental habituation, we began with the examination. The IITC Plantar Analgesia Meter (IITC Life, Sciences, SERIES8, Model 390G) determined the withdrawal delay of the mouse foot from radiant thermal light, placed under the tempered glass and slid across the surface to the exact middle of the plantar’s right hind paw. Each phase was repeated three times per mouse. The device was programmed to stop after 20 s to avoid harming their paws.

### 2.5. Western Blot Analysis

The DRG, SC, Hypo, and PAG tissues were excised to extract protein. Tissues were placed on ice and stored at −80 °C until protein extraction. Total proteins were homogenized in a cold radioimmunoprecipitation (RIPA) lysis buffer containing 50 mM Tris-HCl pH 7.4, 250 mM NaCl, 1% NP-40, 5 mM EDTA, 50 mM NaF, 1 mM Na_3_VO_4_, 0.02% NaN_3_, and 1× protease inhibitor cocktail (Bionovas, FC0070-0001, Taipei, Taiwan). The extracted proteins were subjected to 8% SDS-Tris glycine gel electrophoresis and transferred to a PVDF membrane. The membrane was blocked with 5% non-fat milk in TBS-T buffer (10 mM Tris pH 7.5, 100 mM NaCl, 0.1% Tween 20), and incubated with a primary antibody in TBS-T with 1% bovine serum albumin (BSA) for 1 h at room temperature. Then, a peroxidase-conjugated anti-rabbit antibody, anti-mouse antibody, or anti-goat antibody (1:5000) was used as the appropriate secondary antibody. The bands were visualized using an enhanced chemiluminescent substrate kit (PIERCE) with LAS-3000 Fujifilm (Fuji Photo Film Co., Ltd., Tokyo, Japan). Where applicable, the image intensity of specific bands was quantified using NIH ImageJ software 1.54 h (Bethesda, MD, USA). β-actin or α-tubulin served as internal control.

### 2.6. Immunofluorescence

Mice were euthanized with 5% isoflurane and intracardially perfused with normal saline followed by 4% paraformaldehyde. The brain was immediately dissected and post-fixed with 4% paraformaldehyde at 4 °C for 3 days. Then, the tissues were placed overnight in 30% sucrose for cryoprotection at 4 °C. Next, the brain was embedded in an optimal cutting temperature compound and rapidly frozen using liquid nitrogen before storing the tissues at −80 °C. Frozen segments were cut into 20 mm sections using a cryostat and placed on glass slides. The samples were fixed with 4% paraformaldehyde, and incubated with a blocking solution consisting of 3% BSA, 0.1% Triton X-100, and 0.02% sodium azide for 1 h at room temperature. After blocking, the samples were incubated with the primary antibody (1:200, Alomone, Jerusalem, Israel), CB1 receptor, and pERK, prepared in 1% BSA overnight. The samples were then incubated with the secondary antibody (1:500), 488-conjugated AffiniPure donkey anti-rabbit IgG (H + L), 594-conjugated AffiniPure donkey anti-goat IgG (H + L), and peroxidase-conjugated AffiniPure donkey anti-mouse IgG (H + L), for 2 h at room temperature before fixation with cover slips for immunofluorescence visualization.

### 2.7. CB1 Receptor Agonist and Antagonist Administration

Adult C57BL/6 female mice (*n* = 6) were used for the CB1 agonist or antagonist test. After mice fibromyalgia induction, 1 μL CB1 agonist AEA (Sigma, St. Louis, MO, USA) was administered at the acupoint or i.c.v at 100 μM. Alternatively, 1 μL CB1 antagonist AM251 (Sigma, St. Louis, MO, USA; in 10 µL of saline) was immediately administered at the acupoint or i.c.v at 5 µg. Under light isoflurane anesthesia (1%), AEA and AM251 were administered after fibromyalgia induction.

### 2.8. Chemogenetic Operation

All mice were anesthetized with 1% isoflurane, and then the head was fixed in a stereotaxic device. A 23-gauge stainless cannula was inserted into PVN of Hypo, 0.82 mm posterior, and 0.2 mm lateral of bregma at 0.8 mm below the cortical superficial and fixed to the skull with glue. The inoculant comprised 0.3 μL of viral solution and was injected for more than 3 min through the pump (KD Scientific, Holliston, MA, USA). The injection cannula was kept at PVN for over 2 min to permit the solution to diffuse. A 0.3 μL of hM3Dq or hM4Di DREADD (designer receptors exclusively activated by designer drugs, namely AAV8-hSyn-hM4D(Gi)-mCherry or AAV8-hSyn-hM4D(Gi)-mCherry; Addgene Plasmid #50477 and #50475, Watertown, MA, USA) were injected into the PVN over the course of two weeks. Clozapine N-oxide (CNO; Sigma C0832, St. Louis, MO, USA) was injected to stimulate the DREADD. CNO was thawed in 5% dimethyl sulfoxide (DMSO; Sigma D2650) and diluted with normal saline before an intraperitoneal injection of 1 mg/kg at day 4.

### 2.9. Statistical Analysis

Statistical analysis was performed using the SPSS 21.0 software package. A Shapiro–Wilk test was performed to examine the normality of the results. All statistical data are shown as the mean ± standard error (SEM). Statistical significance was verified using an ANOVA, followed by a post hoc Tukey’s test. *p* < 0.05 indicated statistical significance.

## 3. Results

### 3.1. Intermittent Cold Stress-Induced Fibromyalgia Pain in Mice Reversed by Electroacupuncture

**Figure 1:**
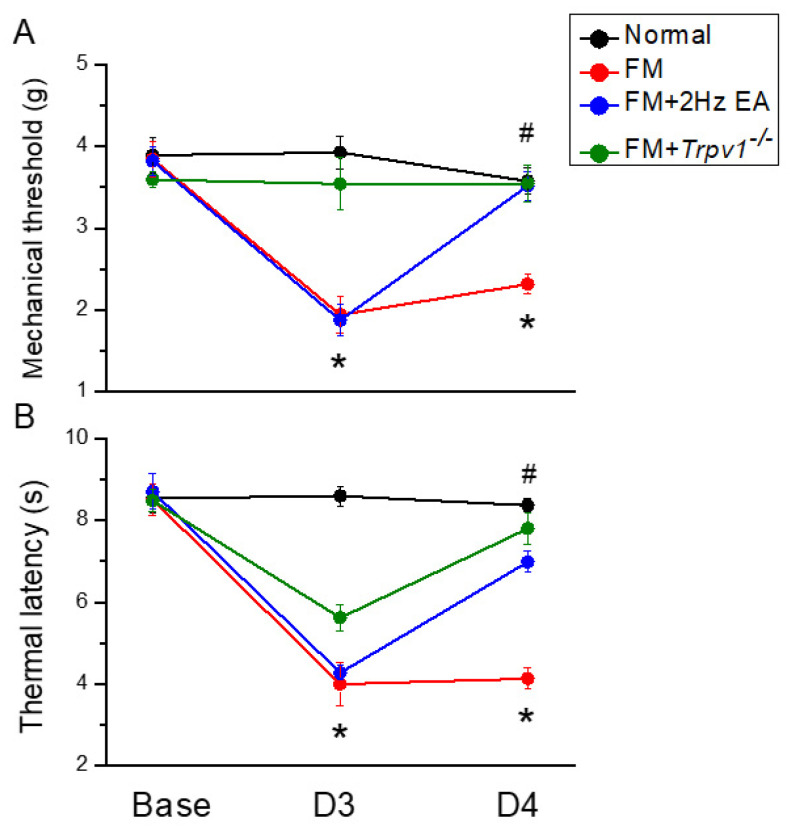
Mechanical and thermal hypersensitivity in the four experimental groups. (**A**) von Frey filament test showing the mechanical threshold; (**B**) Hargreaves test for thermal latency. * *p* < 0.05 = significant differences in comparison to the normal group. ^#^ *p* < 0.05 = significant differences compared to the FM group. *n* = 9.

We measured pain using von Frey and Hargreaves tests to understand how EA attenuates painful sensation in fibromyalgia mice. After applying ICS to four groups of mice for 3 days, their nociceptive response was measured to confirm fibromyalgia pain. Figure 1A depicts the mechanical withdrawal thresholds in the fibromyalgia group, which displayed persistent pain induction compared to animals in the normal group (Figure 1A, red circle, day 4: 2.32 ± 0.12 g, *n* = 9). In contrast, 2 Hz EA significantly alleviated the hypersensitivity of fibromyalgia pain by significantly increasing the withdrawal threshold measured in the von Frey test (Figure 1A, blue circle, day 4: 3.52 ± 0.17 g, *n* = 9). In addition, *Trpv1* gene loss also decreased mechanical hyperalgesia (Figure 1A, green circle, day 4: 2.54 ± 0.23 g, *n* = 9). Furthermore, the Hargreaves test demonstrated that ICS reduced the thermal latency of fibromyalgia mice compared to the normal group, confirming the establishment of fibromyalgia pain (Figure 1B, red circle, day 4: 4.14 ± 0.26 g, n = 9). In contrast, EA diminished thermal hyperalgesia by increasing the thermal latency (Figure 1B, blue circle, day 4: 6.99 ± 0.27g, *n* = 9). A similar effect was observed in *Trpv1^−/−^* mice (Figure 1B, green circle, day 4: 7.80 ± 0.39 g, *n* = 9), suggesting a crucial role of TRPV1 in the development of fibromyalgia pain.

### 3.2. EA Regulated CB1 on TRPV1 Signaling Pathway of Fibromyalgia Pain in the Mouse Peripheral DRG

**Figure 2:**
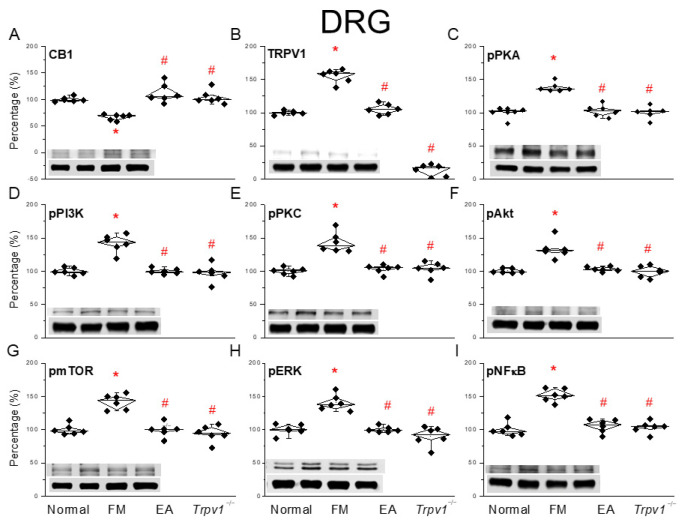
Changing ratio of CB1 and related molecules in the DRG of mice. Black diamonds denote original data distribution. Western blot with four lanes of each protein: Normal, FM, FM + EA, FM + *Trpv1*^−/−^. (**A**) CB1, (**B**) TRPV1, (**C**) pPKA, (**D**) pPI3K, (**E**) pPKC, (**F**) pAkt, (**G**) pmTOR, (**H**) pERK, and (**I**) pNFκB protein levels. * *p* < 0.05 = significant differences in comparison with the normal group. ^#^ *p* < 0.05 = significant differences in comparison with the FM group. *n* = 6.

Next, we used a Western blot analysis to clarify how CB1 affects fibromyalgia pain in peripheral DRG areas. We examined CB1 expression in the murine DRG region. After ICS induction, fibromyalgia pain was observed together with decreased CB1 expression in the DRG, suggesting CB1 downregulation (Figure 2A, * *p* < 0.05, *n* = 6). In contrast, three secessions of EA decreased fibromyalgia pain by increasing CB1 protein levels in the DRG (Figure 2A, ^#^ *p* < 0.05, *n* = 6). In the *Trpv1^−/−^* group, CB1 protein levels were almost normal (Figure 2A, ^#^ *p* < 0.05, *n* = 6). In addition, fibromyalgia pain increased TRPV1 expression, while 2 Hz EA significantly suppressed such overexpression after two sessions. Moreover, TRPV1 expression in the DRG was almost undetectable (Figure 2B, ^#^ *p* < 0.05, *n* = 6). Next, we determined the expression of downstream molecules such as phosphorylated PKA (pPKA), pPI3K, and pPKC, which are all kinases associated with TRPV1. An increased expression of these kinases was observed in fibromyalgia mice compared to the normal group, which was diminished by 2 Hz EA treatment at ST36 acupoints as well as in *Trpv1*^−/−^ mice (Figure 2C–E, ^#^ *p* < 0.05, *n* = 6). In addition, pAkt and pmTOR levels increased in fibromyalgia mice, as did those of pERK and pNFκB (significantly so). EA and *Trpv1* knockout reverted these upregulations (Figure 2F–I, ^#^ *p* < 0.05, *n* = 6).

### 3.3. EA at the ST36 Acupoint Decreased Cold Stress-Induced Fibromyalgia Pain Through CB1-TRPV1 in the SCDH

**Figure 3:**
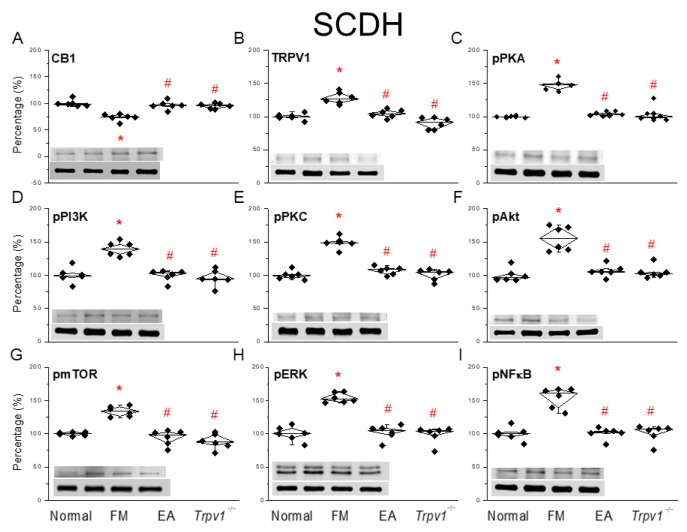
Changing ratio of CB1 and related molecules in the SCDH of mice. Black diamonds denote original data distribution. Western blot with four lanes of each protein: Normal, FM, FM + EA, FM + *Trpv1*^−/−^. (**A**) CB1, (**B**) TRPV1, (**C**) pPKA, (**D**) pPI3K, (**E**) pPKC, (**F**) pAkt, (**G**) pmTOR, (**H**) pERK, and (**I**) pNFκB protein levels. * *p* < 0.05 = significant differences in comparison with the normal group. ^#^ *p* < 0.05 = significant differences in comparison with the FM group. *n* = 6 per group.

As in the previous subsection, we investigated the effects of EA after fibromyalgia induction in the SCDH region. Interestingly, in this region, fibromyalgia pain induction decreased CB1 protein levels (Figure 3A, * *p* < 0.05, *n* = 6). This effect was reverted by 2 Hz EA, which not only relieved fibromyalgia but increased CB1 levels in the SCDH. A similar result was obtained in *Trpv1*^−/−^ mice (Figure 3A, ^#^ *p* < 0.05, *n* = 6). The low TRPV1 expression in normal mice significantly increased in the SCDH of fibromyalgia pain mice (Figure 3B, * *p* < 0.05, *n* = 6); this phenomenon was alleviated by 2 Hz EA treatment and was also observed in *Trpv1*^−/−^ mice (Figure 3B, ^#^ *p* < 0.05, *n* = 6). At the protein level, we observed significantly increased expressions of pPKA, pPI3K, and pPKC in the SCDH of mice in the fibromyalgia pain group. Those increased levels were also attenuated by EA and *Trpv1*^−/−^ mice (Figure 3C–E, ^#^ *p* < 0.05, *n* = 6). Moreover, levels of pAkt and pmTOR, downstream mediators of pPI3K for pain signaling, were increased in the SCDH of fibromyalgia mice. A similar tendency was observed for pERK and pNFκB. These trends were reversed by EA and in *Trpv1*^−/−^ mice (Figure 3F–I, ^#^ *p* < 0.05, *n* = 6).

### 3.4. EA at the ST36 Acupoint Decreased Cold Stress-Induced Fibromyalgia Pain and Regulated CB1-TRPV1 Signaling Pathway in the Hypothalamus

**Figure 4:**
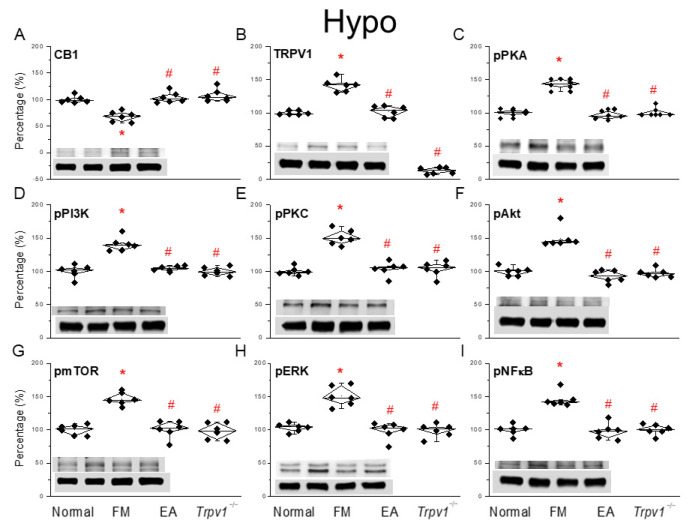
Changing ratio of CB1 and related molecules in the Hypo of mice. Black diamonds denote original data distribution. Western blot with four lanes of each protein: Normal, FM, FM + EA, FM + *Trpv1*^−/−^. (**A**) CB1, (**B**) TRPV1, (**C**) pPKA, (**D**) pPI3K, (**E**) pPKC, (**F**) pAkt, (**G**) pmTOR, (**H**) pERK, and (**I**) pNFκB protein levels. * *p* < 0.05 = significant differences in comparison with the normal group. ^#^ *p* < 0.05 = significant differences in comparison with the FM group. *n* = 6 per group.

After the final behavioral tests on day 5, we collected the total Hypo tissues from all mouse groups and measured their protein expression using a Western blot analysis. CB1 levels were reduced in fibromyalgia pain mice (Figure 4A, * *p* < 0.05, *n* = 6). This indicated a lower antinociceptive effect, which was reversible via EA and *Trpv1* gene loss (Figure 4A, ^#^ *p* < 0.05, *n* = 6). In the Hypo of fibromyalgia pain mice, we observed increased TRPV1 levels, an effect attenuated by EA and observed in *Trpv1*^−/−^ mice (Figure 4B, ^#^ *p* < 0.05, *n* = 6). Similarly to other tissues, levels of TRPV1-associated kinases such as pPKA, pPI3K, and pPKC all increased after ICS-induced fibromyalgia; these effects were alleviated by EA and in *Trpv1^−/−^* mice (Figure 4C–E, ^#^ *p* < 0.05, *n* = 6). Sham EA could not alter the responses. The establishment of the fibromyalgia pain also increased pAkt and pmTOR expression in the Hypo, suggesting its crucial role in pain development. EA treatment or *Trpv1* gene deletion reduced the overexpression of these molecules. Likewise, ICS increased pERK and pNFκB expression. Furthermore, EA or the removal of the TRPV1 protein reliably diminished the overexpression of these factors (Figure 4F–I, ^#^ *p* < 0.05, *n* = 6).

### 3.5. CB1-TRPV1 and Related Factors Were Inhibited in the PAG, an Effect Reversed by EA and Trpv1 Gene Deletion

**Figure 5:**
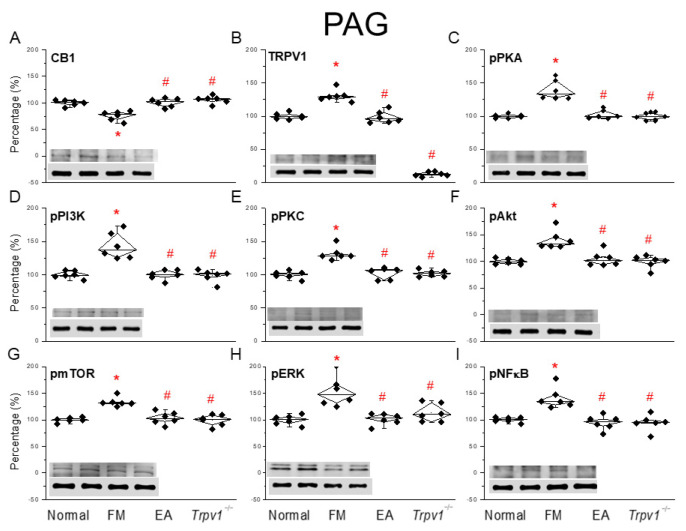
Changing ratio of CB1 and related molecules in the PAG of mice. Black diamonds denote original data distribution. Western blot with four lanes of each protein: Normal, FM, FM + EA, FM + *Trpv1*^−/−^. (**A**) CB1, (**B**) TRPV1, (**C**) pPKA, (**D**) pPI3K, (**E**) pPKC, (**F**) pAkt, (**G**) pmTOR, (**H**) pERK, and (**I**) pNF-κB protein levels. * *p* < 0.05 = significant differences in comparison with the normal group. ^#^ *p* < 0.05 = significant differences in comparison with the FM group. *n* = 6 per group.

Finally, to determine the influence of ICS-induced fibromyalgia pain and EA on fibromyalgia pain signaling in the descending pathway, we subjected the PAG to a protein analysis. We observed a significant decrease in CB1 expression in mice suffering from ICS-initiated fibromyalgia pain (Figure 5A, * *p* < 0.05, *n* = 6), an effect reversed by EA or TRPV1 dysfunction (Figure 5A, ^#^ *p* < 0.05, *n* = 6). In addition, ICS significantly increased TRPV1 expression in the PAG. Such an increase was reduced by EA or *Trpv1* gene deletion (Figure 5B, ^#^ *p* < 0.05, *n* = 6). A comparable trend was perceived in pPKA, pPI3K, and pPKC protein content in fibromyalgia mice compared to normal mice (Figure 5C–E, ^#^ *p* < 0.05, *n* = 6). This effect was significantly reversed by EA and *Trpv1* gene loss. Similar tendencies were observed for pAkt, pmTOR, pERK, and pNFκB (Figure 5F–I, ^#^ *p* < 0.05, *n* = 6).

### 3.6. CB1 and pERK Expression in Peripheral DRG and Central PAG Regions Can Be Modulated by EA or Trpv1 Gene Deletion

**Figure 6:**
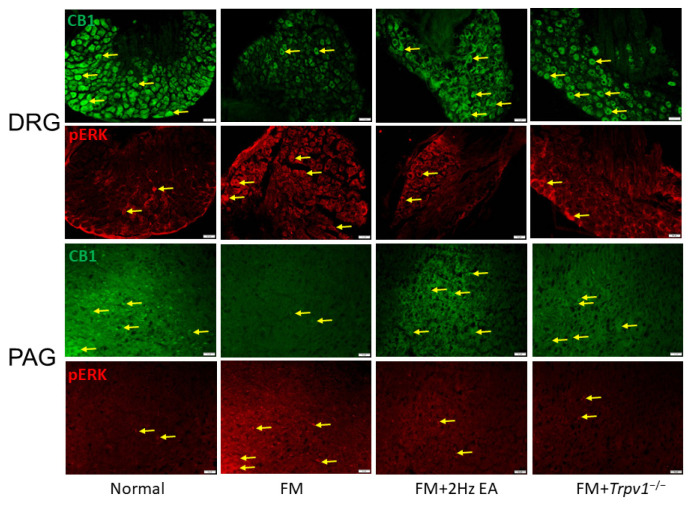
Expression levels of CB1 and pERK in mouse DRG and PAG. Immunofluorescence labeling of CB1 and pERK in mouse DRG and PAG (green and red, respectively). Yellow arrows denote immune-positive signals. White bar = 100 μm. n = 3 per group.

We next examined if ICS could alter CB1 expression in DRG neurons after fibromyalgia induction. Here, we confirmed that fibromyalgia induction significantly decreased CB1 expression in peripheral DRG neurons through CB1-specific immunohistochemistry staining (Figure 6, *n* = 3). Consistently with Western blot findings, animals that received EA or *Trpv1* gene deletion showed increased CB1 expressions. In contrast, pERK levels increased after ICS and were alleviated in EA or *Trpv1* gene deletion groups. Subsequently, we investigated the role of CB1 and pERK in the modulation of ICS-induced fibromyalgia pain in central PAG areas (Figure 6, *n* = 3). Our results revealed that ICS also decreased CB1 expression in the central PAG region. In addition, EA can reverse CBA expression in the PAG regions. A similar tendency was observed in *Trpv1^−/−^* mice (Figure 6, *n* = 3).

### 3.7. EA Diminished Fibromyalgia Directly Targets the CB1 Receptor in the Mice Acupoint or Brain

**Figure 7:**
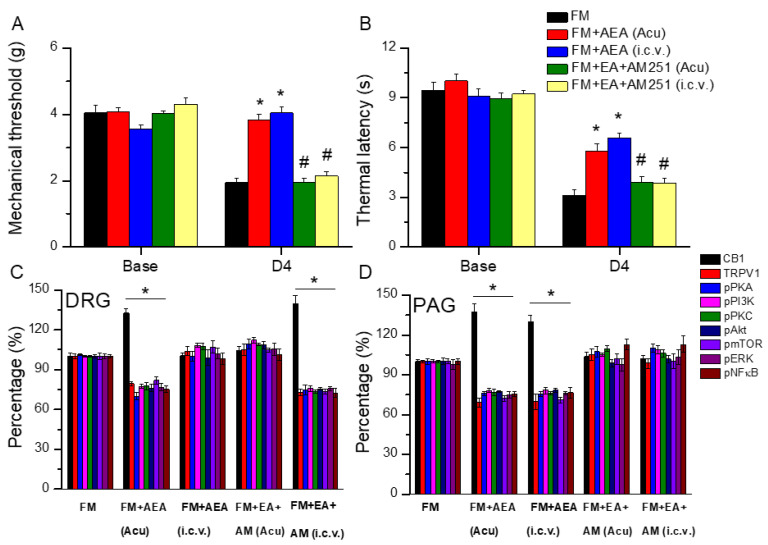
Nociceptive responses in FM mice treated with a CB1 agonist or antagonist. (**A**) Mechanical hyperalgesia and (**B**) thermal pain behavior of FM mice and FM mice treated with a CB1 agonist or antagonist. (**C**) Protein concentrations of CB1, TRPV1, pPKA, pPI3K, pPKC, pAkt, pmTOR, pERK, and pNFκB in mouse DRG. (**D**) Protein concentrations of CB1, TRPV1, pPKA, pPI3K, pPKC, pAkt, pmTOR, pERK, and pNFκB in mouse PAG. Black: FM group; red: FM treated with AEA at acupoint; blue: FM group treated with AEA intracerebral ventricle injection; green: FM treated with EA and AM251 at acupoint; and yellow: FM treated with EA and AM251 at ventricle. * *p* < 0.05 = significant differences in comparison with the FM group. ^#^ *p* < 0.05 = significant differences in comparison with the FM+AEA (Acu) group.

We further examined if EA could regulate CB1 receptor expression to relieve mice fibromyalgia pain. We first confirmed that ICS significantly induced mechanical hyperalgesia in mouse fibromyalgia pain (Figure 7A, day 4: 1.94 ± 0.15 g, *n* = 6). To test our hypothesis that both peripheral and central CB1 activation could reduce mechanical hyperalgesia, we injected the CB1 agonist anandamide (AEA) into the acupoint and intracerebral ventricle. In von Frey filament tests, our data indicated that either acupoint administration (Figure 7A, day 4: 3.82 ± 0.18 g, *n* = 6) or intracerebral ventricle injection (Figure 7A, day 4: 4.04 ± 0.19 g, *n* = 6) significantly attenuated mechanical hyperalgesia. We next used AM251, a CB1 receptor antagonist, to check if EA delivers its analgesic effect through CB1. We demonstrated that EA’s antinociceptive effect was abolished during AM251 antagonism (Figure 7A, day 4, *n* = 6). Similar trends were also observed in thermal latency (Figure 7B, *n* = 6).

After confirming the analgesic effect of EA, we performed Western blotting to further examine changes to CB1 signaling molecules in peripheral DRG regions. Injecting AEA into the acupoint significantly increased CB1 levels in fibromyalgia mouse DRG neurons (Figure 7C, day 4: 132.70 ± 3.41%, * *p* < 0.05, *n* = 6). In contrast, injecting AEA in the central cerebral ventricle did not affect CB1 levels in the DRG (Figure 7C, day 4: 100.50 ± 2.34%, *p* > 0.05, *n* = 6). Similar phenomena were obtained when AM251 was injected into the acupoint (Figure 7C, day 4: 104.22 ± 3.37%, *p* > 0.05, *n* = 6). Further, AM251 intracerebral injection did not affect EA’s effects (Figure 7C, day 4: 139.84 ± 5.85%, * *p* < 0.05, *n* = 6). In contrast, TRPV1, pPKA, pPI3K, and pPKC levels all decreased after the acupoint injection of AEA, but not when AEA was injected into the intracerebral ventricle. This suggests a central role of CB1. Similar results were obtained in EA-treated mice after AM251 acupoint administration. Furthermore, the intracerebral injection of AM251 did not alter the effect of EA on TRPV1 and related molecules. The same trends were obtained in TRPV1 downstream molecules including pAkt, pmTOR, pERK, and pCREB (Figure 7C, day 4, *n* = 6).

To better explore the direct effect of EA on CB1 at a central level, both AEA and AM251 were injected at the acupoint or the intracerebral ventricle after ICS fibromyalgia pain induction. We further used Western blotting to investigate the protein alterations of CB1 signaling in central PAG regions. AEA, injected into the acupoint or ventricle, significantly increased CB1 levels in the PAG neurons of fibromyalgia mice (Figure 7D, day 4, * *p* < 0.05, *n* = 6). In addition, CB1 levels were not altered in the PAG of mice receiving EA and AM251 administration in either the acupoint or ventricle (Figure 7D, day 4, *p* > 0.05, *n* = 6). Conversely, the levels of TRPV1 and related molecules, such as pPKA, pPI3K, and pPKC, decreased following acupoint or intracerebral AEA injection. These factors were not altered in EA-treated mice after AM251 administration. The same trends were obtained for TRPV1 downstream molecules including pAkt, pmTOR, pERK, and pCREB (Figure 7D, day 4, *n* = 6).

### 3.8. Chemogenetic Activation of the Paraventricular Nucleus Initiated Fibromyalgia Pain, and ICS-Induced Fibromyalgia Pain Can Be Further Attenuated by Chemogenetic Inhibition

**Figure 8:**
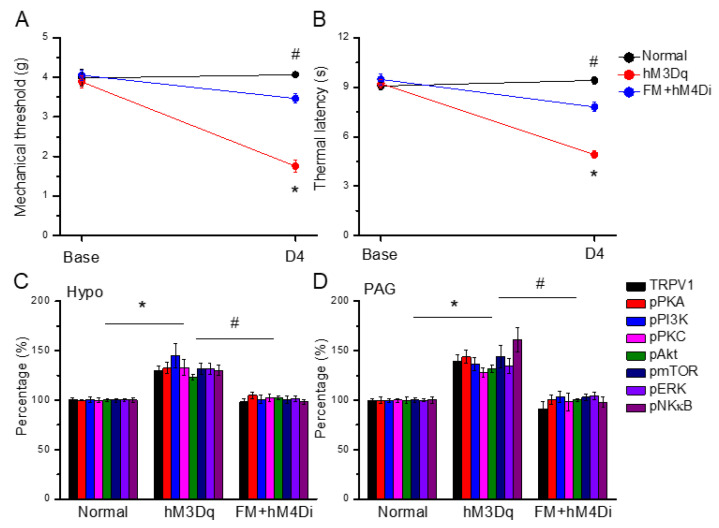
Chemogenetic modulation on mice fibromyalgia pain using TRPV1 and associated molecules. (**A**) Mechanical thresholds and (**B**) thermal latency after CNO activation. (**C**) TRPV1 receptors and related kinases in the Hypo regions of the mice that received CNO stimulation. (**D**) TRPV1 and linked kinases in the PAG regions of the mice that received CNO stimulation. * *p* < 0.05 vs. normal group. ^#^ *p* < 0.05 vs. the hM3Dq group. Mean ± SEM of *n* = 6 mice per group.

Figure 8A shows noteworthy chemogenetic-induced mechanical and thermal hyperalgesia at day 4 (Figure 8A,B, red circles, *n* = 6). In addition, the chemogenetic inhibition of the paraventricular nucleus meaningfully diminished mechanical and thermal hyperalgesia induced by ICS initiation (Figure 8A,B, blue circles, *n* = 6). In addition to a behavioral analysis, we also want to explore changes in TRPV1 pain-related pathways and proteins in the mice Hypo and PAG. Likewise, our results showed that TRPV1 was increased by the chemogenetic activation of the paraventricular nucleus in the mouse hypothalamus and PAG. The pPKA, pPI3K, and pPKC levels were all increased after CNO injection. Similar results were observed for pAkt, pmTOR, pERK, and pNFκB. Furthermore, the chemogenetic inhibition of the paraventricular nucleus significantly attenuated the overexpression of the TRPV1 pathway in Hypo and PAG in fibromyalgia mice (Figure 8C,D, *n* = 6).

## 4. Discussion

To our knowledge, this is the first study to show that ICS-induced fibromyalgia pain in mice can be improved by 2 Hz EA or *Trpv1* gene deletion. Our findings reveal that CB1 protein levels decreased after fibromyalgia induction, while TRPV1 and downstream kinases were increased in the key pain processing regions including DRG, SCDH, Hypo, and PAG. Notably, these alterations could be reversed by 2 Hz EA treatment and TRPV1 knock out, suggesting the pivotal role of CB1-Trpv1 interactions in fibromyalgia pain modulation.

Previous studies have established the essential role of the endocannabinoid system in pain regulation, with CB1 receptors exerting analgesic effects via the modulation of neurotransmission. Cannabidiol is the major nonaddictive component of cannabis, and reportedly interacts with several neurotransmitters to produce its analgesic effects. Notably, De Gregorio et al. reported that acute intravenous cannabidiol injection could reliably decrease the firing rate of serotonergic (5-HT) neurons in the dorsal raphe nucleus. This decrease could be prevented by the administration of 5-HT_1A_ and a TRPV1 antagonist, suggesting its critical involvement in this phenomenon. In addition, cannabidiol appears to induce analgesia mainly through TRPV1, decreasing anxiety through the 5-HT_1A_ receptor [32].

In the peripheral nervous system, the high co-localization of CB1 and Kv1.4 in nociceptive DRG neurons indicates an efficient synergistic action between the two. As the Kv1.4 potassium channel appears to be involved in the control of repetitive discharges at peripheral terminals for pain relief, it may serve as a downstream site of CB1 for the control of presynaptic transmitter release at central terminals [33]. Moreover, Liu et al. proposed that a cannabinoid receptor agonist can significantly diminish the functional current of acid-sensing ion channels in rat DRG neurons. The same result was obtained when using a CB1 receptor antagonist but not a CB2 receptor, suggesting the crucial role of CB1 in the peripheral DRG [34]. A pharmacological description of cannabinoid agonists and antagonists indicated that potent agonists trigger adenylate cyclase and MAPK mainly via CB1 receptors [35]. In this study, we showed that CB1 expression was markedly reduced in fibromyalgia-affected DRG, leading to an overactivation of TRPV1 nociceptive signaling pathways. Conversely, 2 Hz EA or *Trpv1* deletion increased CB1 expression, while suppressing TRPV1 and associated molecules. Additionally, chemogenetic regulation at the PVN reliably modulated fibromyalgia pain through the TRPV1 pathway. This suggests the mechanistic relevance of CB1-TRPV1 signaling, particularly in central pain processing, and highlights EA as an active non-pharmacological intervention for fibromyalgia pain relief.

A recent study reported that cannabidiol could alleviate rat spinal cord injury-induced chronic pain, through dose-dependent reductions in tactile and cold hypersensitivity. This antinociceptive effect was inhibited by the CB1 antagonist AM251, suggesting a possible CB1 mechanism in the SCI pain state [36]. Zhang et al. found that miR-338-5p decreased myelin-associated glycoprotein and glial fibrillary acidic protein, providing neuroprotective effects from spinal cord injury. They next demonstrated that CB1 is the target receptor of miR-338-5p, which can reliably attenuate cell apoptosis and promote neuron survival [37]. In addition, Nerandzic suggested that endogenous lipid precursor N-arachidonoylphosphatidylethanolamine (20:4-NAPE) treatment can inhibit excitatory synaptic transmission in both naive and inflammatory rat models. The application of 20:4-NAPE further activated CB1’s inhibitory effects in naive animals, while TRPV1-mediated mechanisms were involved after inflammation [38]. Jiang et al. found that combined-acupoint EA produced stronger analgesic effects than single-acupoint EA, partially via CB1 and opioid receptors. This suggests a beneficial effect of combined EA through initiating diverse mechanisms from different acupoints [39]. We also demonstrated that 2 Hz EA increased CB1 expression and decreased TRPV1 signaling in the spinal cord, contributing to alleviating fibromyalgia pain.

Beyond spinal mechanism, supraspinal centers such as the Hypo and PAG play pivotal roles in central sensitization. Watanabe et al. determined the stress-induced activation of hypothalamic pain responses, suggesting a mechanistic link between hypothalamic activation and trigeminal nociceptor effectors in migraines [40]. The stress-induced activation of the hypothalamic–pituitary–adrenal (HPA) axis is triggered by the release of a corticotropin-releasing hormone that has been previously reported in the pathogenesis of fibromyalgia. The affective measurement of pain initiated at the SC superficial dorsal horn and ascending pain signals in the brain are associated with unpleasantness including Hypo [41]. Slamberova et al. determined that patients with pain syndromes often present with dysfunction of the HPA axis and increased mast cell infiltration and activation, leading to nociceptive responses. In addition, chronic stress significantly resulted in rodent depression via decreased glutamatergic transmission in the ventrolateral PAG (vlPAG) [42]. A recent study confirmed the role of vlPAG in analgesia [43], while another reported the association of vlPAG excitatory transmission in a murine model of visceral pain [44]. Yin et al. demonstrated that the precise activation of the dmPFC/vlPAG neural pathway via optogenetics caused an analgesic effect in a mouse model of chronic pain. Further suppressing the dmPFC/vlPAG neural circuit resulted in the significant maintenance of chronic pain [45]. Here, our results showed that thermal hypersensitivity was still observed in *Trpv1^−/−^* mice, implying the possible role of other receptors in this model, such as toll-like receptor 4, acid-sensing ion channel 3, or TRPV4 receptors. We showed increased TRPV1 signaling and decreased CB1 expressions in the vlPAG in a fibromyalgia pain mouse model. These phenomena were able to be reversed using 2 Hz EA and *Trpv1* deletion, suggesting the crucial role of TRPV1-CB1 signaling in the Hypo and vlPAG in the supraspinal regulation of fibromyalgia pain.

## 5. Conclusions

**Figure 9:**
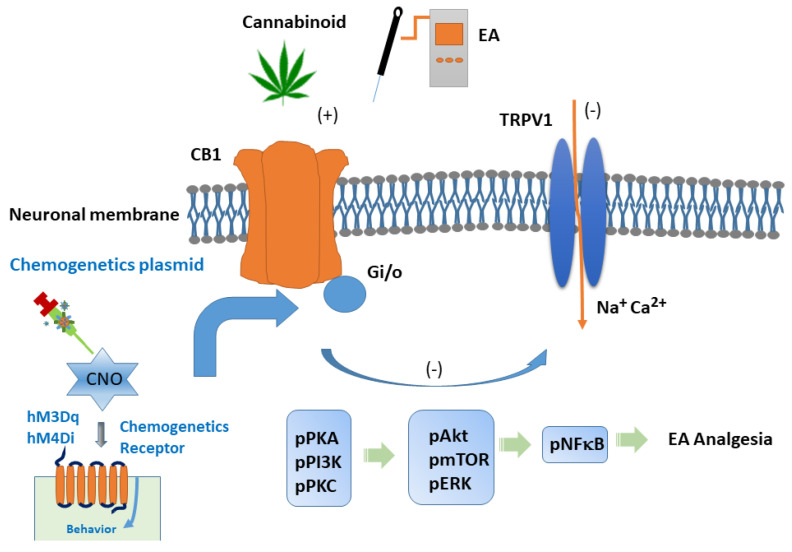
The role of CB1 on TRPV1 signaling pathway in mice fibromyalgia model. Abbreviations: EA = electroacupuncture; TRPV1 = transient receptor potential V1; CB1 = cannabinoid receptor 1; pPKA = protein kinase A; pPI3K = phosphorylated phosphoinositide 3−kinase; pPKC = protein kinase C; pAkt = phosphorylated Akt; pmTOR = phosphorylated mammalian target of rapamycin; pERK = phosphorylated extracellular signal−regulated kinase; pNF-kB = phosphorylated nuclear factor kappa−light−chain−enhancer of activated B cells.

In conclusion, our findings provide precise evidence of the role of 2 Hz EA in the modulation of mice fibromyalgia pain, especially via the CB1-TRPV1 signaling pathway. We characterized the antinociceptive effect of 2 Hz EA on mouse fibromyalgia pain and its underlying cellular mechanisms. Our data revealed that TRPV1 and related kinases were overexpressed in our mouse model of fibromyalgia pain. In contrast, we observed decreased CB1 expression in the DRG, SCDH, Hypo, and PAG. These effects were abolished by EA treatment and also observed in *Trpv1*^−/−^ mice. Novel chemogenetic modulation at PVN attenuated fibromyalgia pain through TRPV1 pathway. Consequently, our results provide indications for the use of EA in fibromyalgia pain and recommend the clinical use of EA. A diagram showing our discoveries is presented in Figure 9.

Our results provide solid evidence for the use of EA and other strategies that induce endocannabinoid signaling in the CB1 for the management of FM pain. A limitation of this study was that we only studied the CB1 pathway in this FM pain model. The clinical investigation of FM patients is necessary to confirm our discoveries; we must try to achieve the clinical application of CB1 as a management objective for FM pain in the future.

## Data Availability

The original contributions presented in this study are included in the article. Further inquiries can be directed to the corresponding authors.

## Abbreviations

The following abbreviations are used in this manuscript:

TRPV1	Transient receptor potential vanilloid 1
*Trpv1*^−/−^	*Trpv1* gene knockout
EA	Electroacupuncture
CB1	Cannabinoid receptor 1
pPKA	Phosphorylated protein kinase A
pPI3K	Phosphorylated phosphoinositide 3-kinase
pPKC	Phosphorylated protein kinase C
pAkt	Phosphorylated Akt
pmTOR	Phosphorylated mammalian target of rapamycin
pERK	Phosphorylated extracellular signal-regulated kinase
pNF-kB	Phosphorylated nuclear factor kappa-light-chain-enhancer of activated B cells
ICS	Intermittent cold stress
PVN	Paraventricular nucleus
DRG	Dorsal root ganglion
SCDH	Spinal cord dorsal horn
Hypo	Hypothalamus
PAG	Periaqueductal gray
